# Non-invasive imaging of ventricular–atrial fistulization secondary to infective rupture of caseous calcification of the mitral annulus

**DOI:** 10.1007/s12574-022-00566-9

**Published:** 2022-02-28

**Authors:** Gabriella Locorotondo, Alessio Angelini, Erica Rocco, Laura Manfredonia, Annalisa Pasquini, Francesca Graziani, Antonella Lombardo

**Affiliations:** 1 Department of Cardiovascular Sciences, Fondazione Policlinico Universitario A. Gemelli IRCCS, Largo A. Gemelli 8, 00168 Rome, Italy; 2 Catholic University of the Sacred Heart, Rome, Italy

In a 78-year-old woman, with history of mitral valve (MV) calcifications and sepsis by *Staphylococcus hominis* with brain micro-embolisms, we diagnosed, by non-invasive imaging, the infective rupture of caseous calcification of the mitral annulus (CCMA), with complete ventricular–atrial fistulization. Trans-thoracic echocardiography (Fig. 1) revealed a gross calcification of the medial commissure of the MV in apical 4-chamber view (panel a), which was confirmed in short-axis view, as involving the posterior mitral annulus from the commissure towards P3 and P2 (panel b); slightly tilting the probe upward from the short-axis view, the medial commissure calcification appeared hypoechogenic in the core, as can be seen in caseous necrosis of calcified annulus (panel c). By colorDoppler mode, huge mitral regurgitation (MR) with jet crossing the CCMA from the ventricular to the atrial side could be detected, both in 4-chamber view (panel d) and short-axis view (panel e). Trans-esophageal echocardiography confirmed and better defined the lesion: in mid-esophageal bicommissural view by black and white mode (Video 1, Fig. 2 panel a), the round-shaped calcification of the medial commissure appeared excavated with frayed and fluctuating edges on the atrial side, which could not be appreciated at transthoracic echocardiography due to calcium shadowing. Two- and three-dimensional colorDoppler mode (Videos 2 and 3), also implemented by contemporary visualization of 2 orthogonal planes (Fig. 2 panel b) showed that MR jet entered the lesion from the ventricular side at the basal portion of the MV and came out on the atrial side at 2 sites, one central and one on the posterior side of CCMA, leading to complete ventricular–atrial fistulization. Scanning the entire MV by colorDoppler mode, revealed MR jet spreading also to the centro-lateral portion, as originating along the coaptation rim (Fig. 2 panel c) and definitively leading to judge MR as severe. Classical volume rendering (Video 4, Fig. 2 panel d) of the atrial side of MV showed the extensive calcification of the posterior mitral annulus, and the large calcified mass of the medial mitral commissure extended to A3. At this level, by  changing the light in the photo-realistic mode, the 2 sites of rupture, on the posterior side of CCMA (Video 5) and centrally (Video 6), could be alternatively and impressively visualized. Computed tomography (CT) showed multiple splanchnic and cerebral embolization, and a large dense calcified mass involving the posterior and anterior mitral annulus. The CT axial slice, crossing the A3–P3 portion of the MV and showing the mitral annulus calcification, is displayed in panel e. As the patient had been recently hospitalized for multifocal pneumonia and brain micro-embolisms, which were initially judged to origin from thrombosis on the CCMA, and only subsequently peripheral blood cultures suggested *Staphylococcus hominis* bacteremia, a positron emission tomography with 18F-fluoro-deoxy-glucose (PET) was performed to confirm active infection of CCMA. To minimize physiological FDG myocardial uptake, the patient was asked to observe a very low-carbohydrate, high-protein and high-fat diet the day before PET imaging and then to fast overnight on the day before imaging, while heparin was not administered before examination. Nevertheless, an intense and diffuse myocardial uptake was found, which was conceivably in keeping with the widespread inflammatory and metabolic activation secondary to the septic state. Unfortunately, it prevented adequate evaluation of the mitral annulus uptake, as can be seen in the corresponding axial slice at positron emission tomography (panel f). Because of several concerns about calcification of mitral annulus, the patient was initially denied from cardiac surgery. However, as her conditions did not stabilize with medical therapy, an extreme attempt of surgical replacement was done. Intraoperatively, a careful descaling of the rear ring was performed, taking care not to weaken the ring itself and not mobilizing the part of calcium infiltrating the ventricle. Finally, a biologic prosthesis was successfully implanted. Unfortunately, the diseased MV did not undergo pathologic examination, but the surgeon’s report stated: “*the mitral valve appears, according to intraoperative echocardiographic description, with erosive lesion as for outcomes of endocarditis in correspondence of A3. In correspondence of the postero-medial commissure, there is presence of a small cavity on the mitral annulus as for evacuated abscess. In correspondence with P3–P2, there are calcifications that infiltrate the ring and part of the basal portion of the heart muscle*”.

**Fig. 1:**
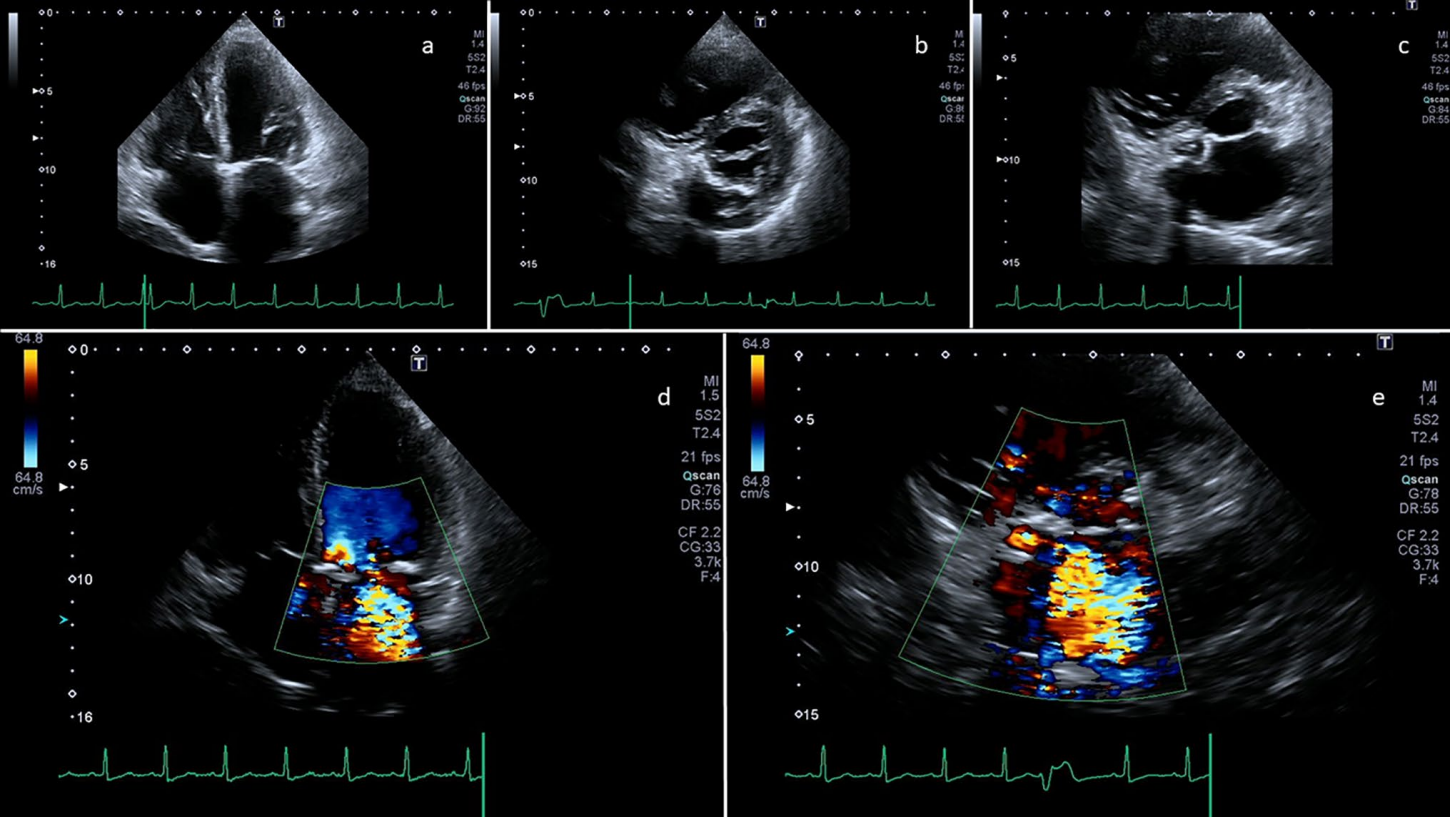
Trans-thoracic echocardiography showing CCMA in black and white mode in 4-chamber view (**a**) and in short-axis view with ultrasound beam directed at the level of the mitral valve plane (**b**); black and white off-axis slice, intermediate between short axis at the level of mitral valve and short axis at the level of aortic valve (**c**) obtained by tilting upwards the probe; colorDoppler mode in 4-chamber view (**d**) and modified short-axis view (**e**)

**Fig. 2:**
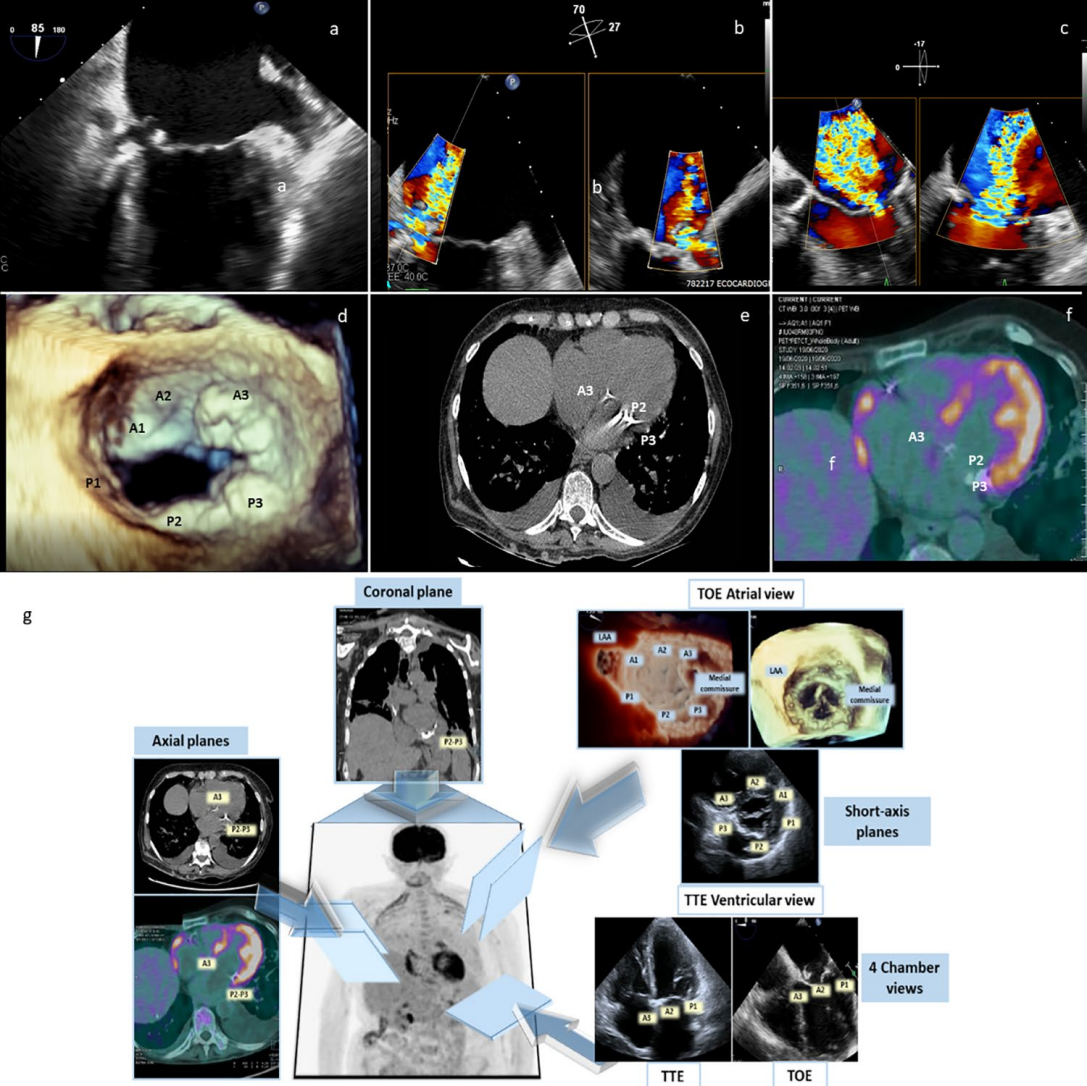
Trans-esophageal echocardiography showing CCMA in black and white mode in 2 chamber view (**a**) and X-Plane visualization of mitral regurgitation by colorDoppler mode (**b**, **c**); three-dimensional visualization of the atrial side of mitral valve by classical volume rendering (**d**) during diastole; computed tomography axial slice (**e**) and corresponding image of diffuse uptake of fluoro-deoxy-glucose at positron emission tomography (**f**); **g** shows a schema illustrating representative multi-modality imaging approach for the mitral valve assessment

Caseous calcification of the mitral annulus (CCMA) rarely extends to the medial commissure and the anterior annulus of mitral valve (MV), and may be associated with MV dysfunction, small erosion and systemic embolization of caseous material. *Staphylococcus* spp. may infect CCMA, because of their specific affinity for osteoblasts [1]. Usually, CCMA fistulization is seen intraoperatively: in only one case, it has been described by echocardiography, but without infection [2]. Echocardiography should always enter the diagnostic workflow of complicated CCMA, but every cardiac imager should familiarize with the different visualizations of CCMA, as provided by different imaging tools. Thoracic CT scan without contrast agents is frequently used in clinical practice in patients potentially candidate to cardiac surgery, because extensive calcification of ascending aorta or valve apparatus may challenge the surgical strategy. On the contrary, while PET is recommended by guidelines [3] in prosthetic and cardiac device-related endocarditis, in native valve endocarditis, evidences about its diagnostic role are weak, with low sensitivity and high specificity [4]. Nevertheless, the best tailoring of treatment should rely on planning based on the most accurate imaging diagnosis. For educational purpose, representative multi-modality imaging visualization of the mitral valve is displayed in panel g.

## Supplementary Information

Below is the link to the electronic supplementary material.

Supplementary file1 (MP4 2138 KB)

Supplementary file2 (MP4 7500 KB)

Supplementary file3 (MP4 218 KB)

Supplementary file4 (MP4 671 KB)

Supplementary file5 (MP4 217 KB)

Supplementary file6 (MP4 109 KB)
